# Supplementary material for: Unraveling Nitrogen Fixing Potential of Endophytic Diazotrophs of Different Saccharum Species for Sustainable Sugarcane Growth
Source: Int J Mol Sci. 2022 Jun 2;23(11):6242. doi: 10.3390/ijms23116242 (PMC9181200; doi:10.3390/ijms23116242)
Supplement: Supplementary file 1 [file ijms-23-06242-s001.zip › ijms-1694952-supplementary.pdf]

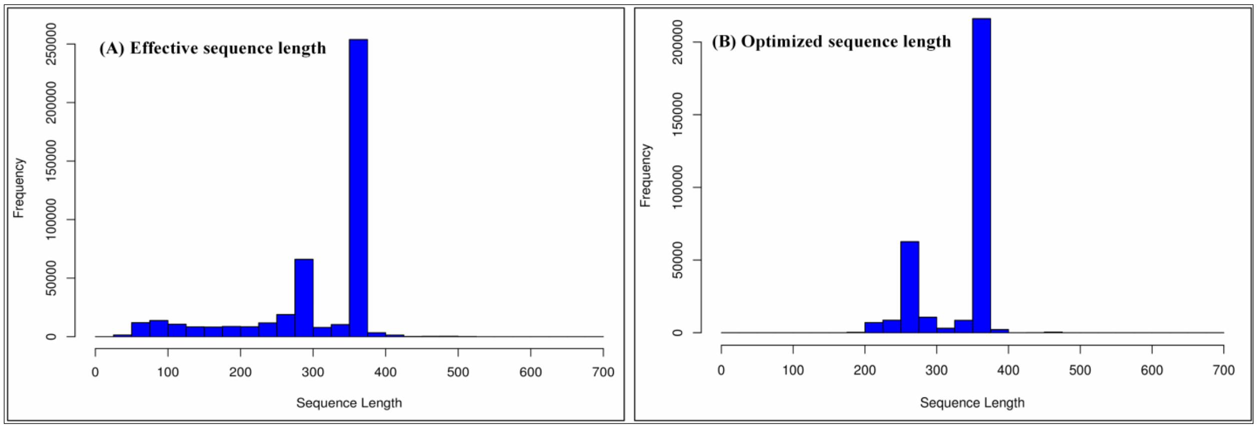

Figure S1. Statistical distribution of sequence length **(A)** Effective sequence length **(B)** Optimize sequence length.

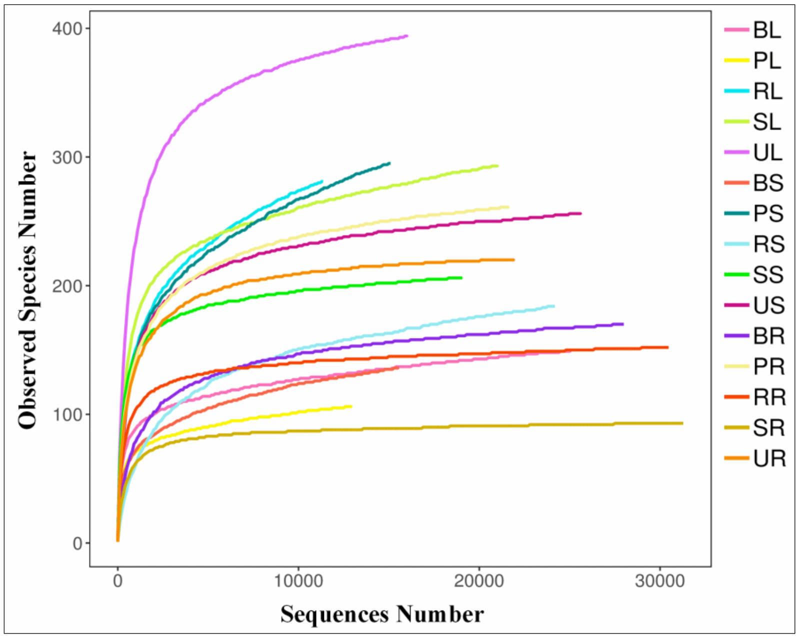

Figure S2. Dilution curve Rarefaction-curve to compare the richness of species in each sample.

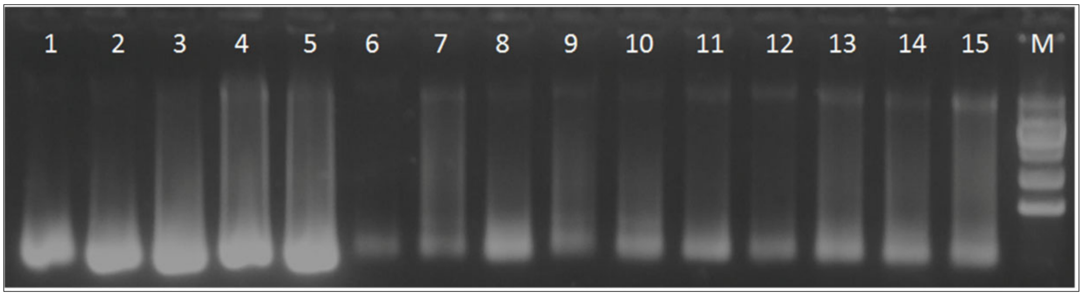

Figure S3. Genomic DNA isolation of different tissues of five sugarcane species. Marker (M) is DL9000. The top to bottom strip is 9000bp 5000bp 3000bp 2000bp 1000bp 500bp, the loading is 3uL, the bright band is 30ng/uL, and the other bands are 10ng/uL. 1. BL; 2. PL; 3. RL; 4. SL; 5. UL; 6. BS; 7.PS; 8. RS; 9. SS; 10. US; 11. BR; 12. PR; 13. RR; 14. SR 15. UR.

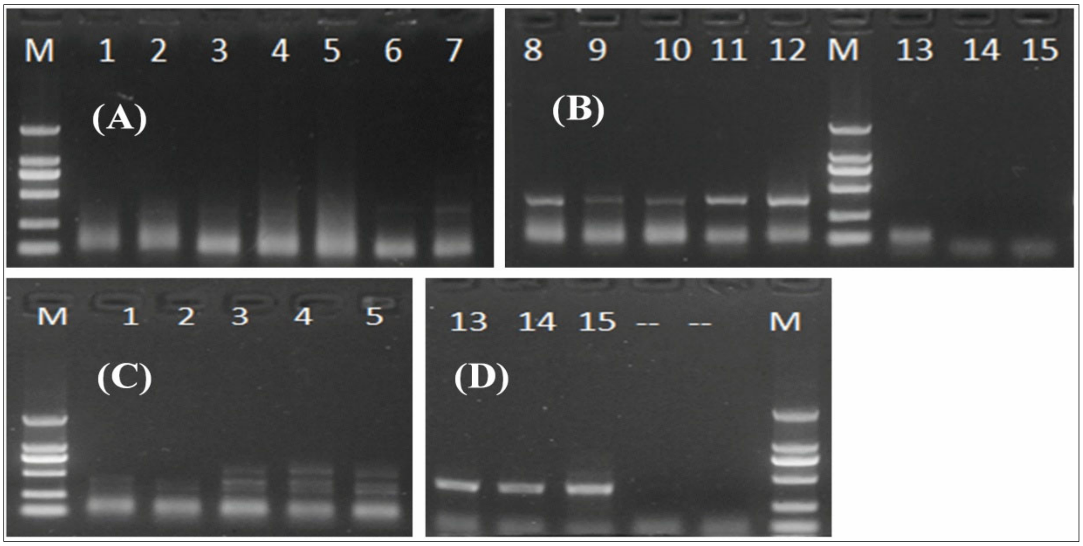

Figure S4. Electrophoresis results of first-round PCR amplification of *nifH* gene. Marker (M) is DL2000. The top to bottom strip is 2000bp 1000bp 750bp 500bp 250bp 100bp, the loading is 3uL, the bright band is 30ng/uL, and the other bands are 10ng/uL. 1. BL; 2. PL; 3. RL; 4. SL; 5. UL; 6. BS; 7.PS; 8. RS; 9. SS; 10. US; 11. BR; 12. PR; 13. RR; 14. SR 15. UR.

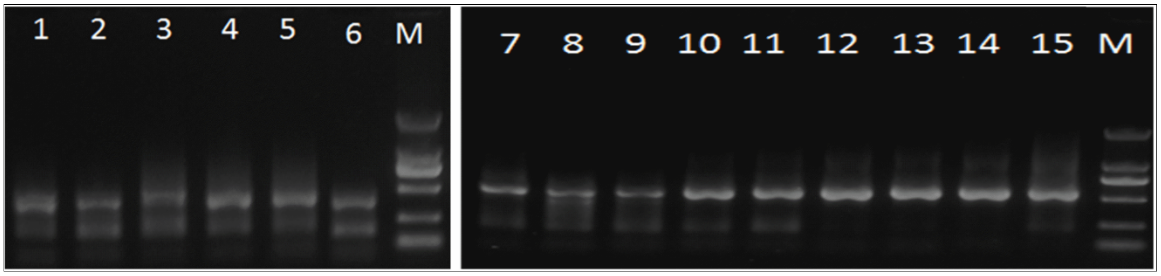

Figure S5. Electrophoresis results showed after second-round PCR amplification of *nifH* gene bands. Marker (M) is DL2000. The top to bottom strip is 2000bp 1000bp 750bp 500bp 250bp 100bp, the loading is 3uL, the bright band is 30ng/uL, and the other bands are 10ng/uL. 1. BL; 2. PL; 3. RL; 4. SL; 5. UL; 6. BS; 7.PS; 8. RS; 9. SS; 10. US; 11. BR; 12. PR; 13. RR; 14. SR 15. UR.
